# Effects of COVID-19 on Visitor Intentions to Attend Personal Interpretation Programs in the Provincial Parks of Alberta, Canada

**DOI:** 10.1177/10925872231217489

**Published:** 2023-12-18

**Authors:** Glen Hvenegaard, Elizabeth Halpenny, Clara-Jane Blye, Katherine Corrigan

**Affiliations:** 1 University of Alberta – Augustana Campus, Camrose, AB, Canada; 2 University of Alberta, Edmonton, AB, Canada

**Keywords:** environmental interpretation, parks and protected areas, survey research, North America

## Abstract

Research has examined COVID-19’s impacts on parks, but little research has studied the pandemic’s impact on in-person interpretation. Based on responses from 431 visitors to Alberta’s provincial parks before and during the pandemic, this paper investigates how the pandemic affected visitor intentions to attend personal interpretation programs. Intentions to attend programs decreased after the pandemic started, but were greater for respondents who had attended programs the previous season. Key reasons for not attending programs were not to become infected and not to infect others. Intentions to attend programs were greater for males than females, and greater for respondents with an increased education and a larger household income. Despite pandemic concerns, 53% of respondents said that programs should be offered, with highest support for amphitheater shows, followed by guided hikes, point duties, and family events. Park managers should clearly communicate the benefits and safety measures employed for interpretation programs.

## Introduction

COVID-19 first emerged as a significant global threat in December 2019 in Wuhan, China (Sharma et al., 2020). On March 11, 2020, the World Health Organization declared COVID-19 to be a Global Pandemic (Sharma et al., 2020). In mid-March 2020, Health Canada advised travelers to avoid all non-essential travel and mandated all travelers entering the country from international travel to quarantine for 14 days.

At first, visitation to parks around the world declined, largely due to the loss of international visitors, but later, domestic visitation increased and, in many cases, surpassed visitor levels from pre 2020 (Souza et al., 2021; Templeton et al., 2021; Wipf et al., 2023). The COVID-19 pandemic led many parks to impose safety processes to reduce the spread of the virus (McGinlay et al., 2020; Smith et al., 2021). Due to various public safety measures, the pandemic impacted park revenues, budgets, staffing, and livelihoods of those living in and around nearby communities (Hockings et al., 2020; Miller-Rushing et al., 2021; Spenceley et al., 2021). These measures also affected the delivery of programs such as in-person interpretation and environmental education that typically occurs in parks and protected areas.

Many parks use personal interpretation, or interpretation that occurs through in-person interactions, to engage the public and foster stewardship amongst visitors. In fact, many park visitors expect opportunities for personal interpretation (Schliephack et al., 2013). Moreover, personal interpretation can be more effective than non-personal interpretation in achieving park goals, such as satisfaction for visitors to Kruger National Park, South Africa (Roberts et al., 2014) or increased awareness and support among visitors for invasive species management at Cumberland Island National Seashore, Georgia, USA (Sharp et al., 2012).

While the impacts of COVID-19 on outdoor recreation and parks have received considerable research attention, researchers know little about the impacts COVID-19 on visitor intentions to participate in personal interpretation activities. Potential decreases in attendance at personal interpretation programs are concerning to park managers for multiple reasons. Park visitors might miss enjoyable activities, educational opportunities, memorable experiences, and increased connections to place (Hvenegaard & Shultis, 2016). At the same time, park managers might miss chances to improve visitor attitudes and behaviors to support park values (Hvenegaard et al., 2023).

## Literature Review

By March 2020, many parks closed facilities in response to COVID-19 (Jacobs et al., 2020). These closures resulted in a significant decrease in visitation, affecting visitor experiences, conservation revenues, business sustainability, local livelihoods, and wildlife crime (Spenceley et al., 2021). Another COVID-19 challenge resulted from canceled or greatly reduced interpretive programs and other educational activities in parks (Corrigan & Hvenegaard, 2021; Collins et al., 2020; McGinlay et al., 2020). South Africa’s SANParks, for example, lost 50,000 educational visits between April and June 2020 (Smith et al., 2021). However, as the pandemic progressed, many parks allowed some visitation and programing, accompanied by safety procedures (McGinlay et al., 2020; Spenceley et al., 2021). Zion National Park (USA), for example, adapted with reduced spaces available on shuttles, increased sanitation, plexiglass barriers, re-formatted events from indoors to outdoors, generous cancellation policies, and reduced food services (Templeton et al., 2021).

In many places, domestic visitation to parks and natural areas increased during the pandemic (Tyrväinen et al., 2021), especially near large urban areas (Ma et al., 2021). Notwithstanding any previous levels of overtourism, the increase in visitation due to COVID-19 was challenging for many managers due to visitors not following social distancing guidelines, new visitors unfamiliar with appropriate park behaviors, and conflicts between different user groups (McGinlay et al., 2020; Templeton et al., 2021). To tackle the subsequent issues of overcrowding, irresponsible users, parking and traffic concerns, and social distancing, many parks implemented or enhanced management strategies. For example, parks increased signage and social media campaigns, capped the number of visitors (entering parks, joining guided tours, and entering facilities), increased the number of rangers, and established one-way hiking on popular trails (McGinlay et al., 2020). Other measures included online educational programs (instead of in-person programs), enhanced cleaning regimes, mandatory digital payment to reduce physical contact, and distribution of hand-sanitizer (Corrigan & Hvenegaard, 2021; McGinlay et al., 2020). Visitors also engaged in various social distancing behaviors (Taff et al., 2022).

Before these pandemic-induced changes to visitation, behavior, and management, many visitors wanted parks and wildlife ecotourism experiences to offer interpretive services (Lück, 2015; Schliephack et al., 2013) and supported interpretive programs to fulfill the mandate of parks (Hvenegaard, 2017). In fact, visitor satisfaction with the interpretation system and educational programs at Banff National Park, Canada increased from before to during the pandemic (C. D. Geng et al., 2023). Even before the pandemic, some park visitors preferred guided (or personal) interpretation to non-guided interpretation (Phan & Schott, 2019; Roberts et al., 2014). Visitors are motivated to attend personal interpretation programs for various reasons, such as nature appreciation, access to nature experiences, socialization, relaxation, enjoyment, escape, learning, challenge, reflection, skill development, and novelty (e.g., Hvenegaard, 2017; Cook et al., 2021; S. Huang et al., 2020). At the same time, visitors face various barriers (e.g., perceptions of danger and safety, time, knowledge, and financial) to participation and have many reasons for not attending interpretation programs (Hvenegaard, 2017). Prior to the COVID-19 pandemic, visitor assessments of danger in a park related to visitor perceptions of management quality, personal choices, skills, and safety (Gstaettner et al., 2022). Researchers know little about how the pandemic affected visitor intentions to participate in interpretive programs.

While researchers have shown that many park visitors felt less safe during the pandemic than before the pandemic (Budruk et al., 2021), little is known about the underlying factors and processes that inform those feelings. However, some research on decision-making in outdoor recreation participation can help understand decisions for visitors to attend personal interpretation programs. For example, during the pandemic, the strongest predictors of risk perception for ecotourists in national parks were media exposure and health and safety information (Samdin et al., 2022). In addition, personality factors (e.g., risk attitude, impulsiveness, and prior knowledge) and environmental factors (e.g., social environment and natural environment) affected risk perception during COVID-19 (Panno et al., 2021; Xu et al., 2022). In a different study, visitors who perceived the greatest threats from COVID-19 undertook activities that were more varied during their travels and had more experience at the site than visitors who perceived fewer threats (Kim et al., 2022).

Overall, tourists who perceived high levels of risk from COVID-19 had lower intentions to travel (Agyeiwaah et al., 2021). We would expect a similar pattern for intentions to participate in personal interpretation programs. Participation in some outdoor recreation pursuits (e.g., backpacking, camping, and outdoor climbing) decreased due to COVID-19, but increased in other activities (e.g., birding, wildlife viewing, gardening, and outdoor running; Rice et al., 2020). The ability to ensure social distancing was a key factor in choosing outdoor activities and respondents strongly agreed that they did not want to expose themselves to COVID-19. Nevertheless, once the threat of COVID-19 became minimal, outdoor recreationists said that they were very or extremely likely to return to their preferred recreational behaviors and patterns (Rice et al., 2020).

Some demographic variables affected intentions to participate in outdoor recreation activities. For example, Weber et al. (2002) found women were more risk-averse when assessing financial, health/safety, recreational, and ethical aspects of an issue, but not those issues related to social risk. Amid the pandemic, the importance of nature for reducing stress was greater for females than males (Tyrväinen et al., 2021). Among birders, females were more likely to restrict their activities than males during the pandemic (Randler et al., 2020). Older adults had higher anxiety levels about COVID-19 than younger adults (likely because older adults are at a higher risk of severe outcomes; Pearman et al., 2021). Perceived risk also varied by country and culture (T.-Y. Huang et al., 2021; Shen et al., 2021; Xu et al., 2022).

The body of research on COVID-19 impacts on outdoor recreation in general is growing, but researchers know little about how COVID-19 affects visitors’ intentions to participate in park services, such as personal interpretation programs. Therefore, this paper reports on research that examined how visitor intentions to participate in personal interpretation programs in Alberta’s provincial parks were affected by the COVID-19 pandemic. We chose Alberta because its park interpretation programs have had a long history, were operating a variety of interpretation programs, and we had an established research program in the park system. We asked three research questions:

1. Did visitor intentions to participate in personal interpretation programs change from before the pandemic to after the pandemic?
2. Did recent participation in personal interpretation programs increase visitor intentions to participate in, and support the provision of, personal interpretation programs during the pandemic? and
3. Did demographic variables affect intentions to participate in, and support the provision of, personal interpretation programs during the pandemic?

## Methods

### Study Site

Alberta Parks operates 473 sites (covering about 4.55 million ha), of which 76 are provincial parks (Alberta Parks, 2022). The agency’s vision is to “inspire people to discover, value, protect, and enjoy the natural world and the benefits it provides for current and future generations” (Government of Alberta, 2009, p. 3). Each year, these sites receive about 8.76 million visits, including about 1.92 million overnight camping visits (Alberta Parks, 2016). Normally, during late June to early September (the highest visitor use periods), 11 parks in the system offer a variety of personal interpretation programs and services such as guided hikes, interpretive talks, bus tours, outdoor theater shows, point duties, and roving interpretation. There is free day-use entry to provincial parks and most interpretive programs are free, except for a few bus tours and specialized guided hikes. Before the pandemic, public interpretation programs, plus environmental education programs for schoolchildren, reached about 450,000 people per year (Alberta Parks, 2016). At the onset of the pandemic, Alberta Parks closed all sites in March 2020, but reopened in May 2020. At first, parks focused on virtual interpretation opportunities, but by July 2020, some parks offered revised forms of personal interpretation (Corrigan & Hvenegaard, 2021).

### Data Collection

Prior to the survey, we conducted a pilot study with a convenience sample of 15 experts and potential park visitors to test for validity and user-friendliness. We then collected data in two stages. In stage 1 (the initial sample), as part of a separate study on the outcomes of interpretation, during June–September 2019, we visited all 11 parks that offered personal interpretation 1 to 2 times. On weekdays, weekend days, and holidays, we collected visitor data using on-site, self-administered surveys. We approached overnight park visitors at various locations in the parks, such as trailheads, outdoor theaters, visitor information centers, and campsites. We used a stratified, convenience-based sampling strategy randomized by interviewing the next available camper as long as we did not observe potential respondents occupied by other activities that would preclude them from an interview (e.g., setting up camp). We used Android tablets (with the Qualtrics offline survey application) and paper-based survey questionnaires to collect all data on-site.

Study questions included demographic and trip information. We received details about birth year (converted to age), gender (six options in addition to “other” and “prefer not to respond”), formal education (1 = elementary school to 6 = graduate degree), household income (1 = <50,000 CAD to 4 = >150,000 CAD), and whether respondents had participated in an interpretive program. Respondents were then categorized based on whether or not they attended a personal interpretation program during their visit to the park. Last, we asked non-attendees how likely they were to attend an interpretive program on their visit (1 = definitely not to 7 = definitely). We did not ask attendees this question because they had attended a program.

In stage 2, after COVID-19 became widespread in Alberta, we contacted all previous respondents during July–August 2020, for a follow up survey through email or telephone, with one reminder, to obtain a subsample of online responses. First, we asked respondents how often they had attended an interpretation program in the past 12 months (excluding the interpretation program from the 2019 survey; 1 = never to 7 = every time). We also asked respondents, in light of COVID-19, if they planned to attend a personal interpretation program in a provincial park during the summer of 2020 (yes/no). We asked, in light of the COVID-19 pandemic, whether personal interpretation programs should be offered in Alberta’s provincial parks in 2020 (1 = not at all to 7 = most definitely). We also asked if five types of interpretation programs should be offered in the summer of 2020 (yes/no). For respondents not intending to attend a program in 2020, we asked how applicable eight potential reasons were for not attending (e.g., avoid infection, avoid infecting others, and access online program instead; 1 = not applicable to 7 = very applicable). At the end of the survey, we asked respondents to provide any other comments; respondents typed in their comments online.

It is worth noting that we collected data about intentions to participate in personal interpretation programs between the first and second waves of the pandemic and before vaccines were available in Alberta. Now that the pandemic has undergone multiple waves and opportunities to vaccinate are available, visitors’ intentions may have changed (Scandurra et al., 2023).

### Data Analysis

Our dataset included only those respondents that took part in both stages. We used SPSS 28 for data analysis. For pairs of dichotomous variables, we used analyses of cross tabulation tables, reporting *X*^2^-values. For dichotomous independent variables with quantitative dependent variables, we used paired samples *t*-tests, reporting Cohen’s *d* to highlight the size effect or impactfulness of each significant relationship (effect sizes are small (*d* = 0.2), medium (*d* = 0.5), and large (*d* = 0.8) (Cohen, 1988)). We report statistical values whenever relationships are significant at *p* < .05. For the additional open ended comments, we used NVivo12 to conduct a simple thematic analysis that involved reading over all comments, identifying categories, placing comments in categories, reviewing these categories among researchers, and finalizing the category list (Braun & Clarke, 2012). We then identified and reported comments that represented those categories.

## Results

### Sample Characteristics

In 2019, we received 875 responses, with a response rate of 90%. The 2020 sample (again, a subsample from the 2019 group) produced 431 responses, with a response rate of 57%. There was a roughly even split between attendees and non-attendees of programs (Table 1). There was no difference among attendees and non-attendees for mean age (*t* = −0.253, *df* = 426, *p* = .400, *d* = 0.02), gender (*X*^2^ = 7.252, *df* = 3, *p* = .064), and household income (*t* = 0.808, *df* = 350, *p* = .210, *d* = 0.01). Previous program attendees had a higher educational attainment than non-attendees did (ordinal scale: *t* = 3.761, *df* = 428, *p* < .001, *d* = 0.36; and categorical scale: *X*^2^ = 18.547, *df* = 5, *p* = .002).

**Table 1.** Demographic Characteristics of 2020 Sample, Broken Down by Attendees and Non-attendees.

	Previous attendees	Previous non-attendees
Number of respondents	227	204
Mean age (years)	46.1	46.4
Gender (%)		
Male	30.8	37.7
Female	67.0	57.4
Other, no answer	2.2	4.9
Education (mean on ordinal scale)	4.4	4.0
Education (% in categories)		
Less than diploma	0.4	0.0
High school diploma	7.1	14.7
College diploma or apprenticeship	24.8	29.9
Some university	8.0	11.8
Bachelor’s degree	35.8	31.9
Graduate degree	23.9	11.8
Household income (mean on ordinal scale)	3.0	2.9
Household income (% in categories)		
<50,000 (CAD)	9.7	7.8
50,000–99,999	19.4	30.7
100,000–149,999	36.6	27.7
>150,000	34.4	33.7

### Intentions to Attend Before and During the Pandemic

Before the pandemic (stage 1), we asked non-attendees how likely they were to attend an interpretive program on their visit. Overall, the mean score was 3.6 (*SD* = 1.96), with 33.8% indicating probably or higher. In stage 2 (which occurred during the pandemic), all respondents were asked if they were planning to attend a program during the summer of 2020; only 41.3% said yes or most definitely. Comparing the groups, 55.8% of 2019 attendees said yes or most definitely, whereas 24.9% of 2019 non-attendees said yes or most definitely (Table 2).

**Table 2.** Past Attendance, Future Intentions to Attend, and Support for Personal Interpretation Programs in 2020 From Previous Attendees and Non-attendees.

	Total (%)	Previous attendees	Previous non-attendees
How often did you attend a personal interpretive program in the past 12 months?			
Never	26.0	9.5	46.0
Rarely	14.5	14.0	15.0
Occasionally	21.7	24.8	18.0
Sometimes	15.4	19.0	11.0
Frequently	6.1	7.9	4.0
Usually	9.3	13.6	4.0
Every time	7.0	11.2	2.0
Do you plan to attend a personal interpretation program in a provincial park during the summer of 2020? (% yes)	41.3	55.8	24.9
Do you believe that programs should be offered during the 2020 season?			
No or not at all	17.8	10.4	25.9
Not really	11.9	7.6	16.6
Maybe	17.3	14.7	20.2
A little bit	11.9	10.9	13.0
Yes or most definitely	41.1	56.4	24.4
Which types of programs should be offered? (% yes)			
Guided hike	52.5	48.1	57.7
Amphitheater show	56.9	66.0	45.8
Point duty (pop-up display)	36.6	37.2	35.8
Bus tour	4.7	6.4	2.5
Family event	30.8	36.4	23.9

For those who did not plan to attend a program, the most common reasons related to fear of becoming infected, not wanting to infect others, group size, and using written sources instead (Table 3). Attendees were more concerned about their travel party’s size being too large to take part (Table 3; *t* = 4.774, *df* = 140, *p* < .001, *d* = 0.91) and being not physically able to take part (*t* = 1.874, *df* = 111, *p* = .032, *d* = 0.48), with medium to large differences observed. Non-attendees were more concerned about not maintaining physical distancing (*t* = −2.136, *df* = 110, *p* = .017, *d* = 0.51). Other reasons showed no differences between previous attendees and non-attendees.

**Table 3.** Mean Rating of Reasons Not to Attend a Personal Interpretation Program During 2020, as Provided by Previous Attendees and Non-attendees.

Reason	% Rating 5–7	Total mean score	Previous attendees	Previous non-attendees
I do not want to get infected	69.0	5.37	5.32	5.40
I do not want to infect others	66.2	5.29	5.44	5.11
My group is too large to attend	59.6	4.96	5.38	3.79
I will use written sources of information instead	50.0	4.94	4.39	5.29
I am not physically able to take part	58.9	4.86	5.00	4.11
I cannot maintain physical distancing (2 m apart)	50.4	4.68	3.95	4.86
I was able to access virtual or online programing	55.8	4.63	5.05	4.43

When asked about which types of interpretation programs should be offered in 2020, respondents gave moderate support to amphitheater shows and guided hikes, slightly less support to point duties and family events, and very little support to bus tours (Table 2). Previous attendees were more supportive than non-attendees for amphitheater shows and family events, but non-attendees were more supportive of guided hikes (Table 2). Some previous respondents recognized efforts given to ensure safe in-person programs. For example, even though one respondent said, “*programs are challenging with COVID-19*,” another respondent said, “*If we can maintain physical distancing between groups, it is important to use this opportunity of people getting outdoors to teach them more about nature & protecting the environment*.” A final respondent summarized efforts by saying that “*I am sure there are safe ways to continue interpretive programming and encourage parks to get creative!*”

### Recent Participation and Intentions to Attend or Support

When we asked respondents in 2020 how often they had attended programs in the past 12 months, attendees in 2019 were much more likely to have attended than non-attendees in 2019 (Table 2; *X*^2^ = 89.730, *df* = 6, *p* < .001). In addition, attendees from 2019 were twice as likely to attend a program in 2020 than non-attendees from 2019 were (Table 2; 55.8% vs. 24.9%; *X*^2^ = 42.495, *df* = 1, *p* < .001). In terms of support, previous attendees were much more likely to support (indicating yes or most definitely) offering interpretive programs in 2020 than non-attendees were (Table 2; 56.4% vs. 24.4%; *X*^2^ = 55.446, *df* = 6, *p* < .001). Among other comments offered, some respondents indicated strong support for offering in-person interpretation programs in 2020 (Table 2). One respondent said, “*the interpretive programs are extremely valuable and should absolutely be continued in the future. The amphitheater shows are one of the best reasons to camp in Alberta parks.”* Another respondent looked to the future in saying “*the interpretive programs are an additional highlight that I hope do not ever get canceled.”* Past attendees expressed appreciation for how staff delivered programs safely during the pandemic. For example, one respondent said, “*Huge shout out to the parks staff who did an awesome job fitting people in, and ensuring the audience maintained social distancing while watching!”* Another respondent said, “*the interpretive staff are doing a great job this year with all the regulations.”*

### Demographic Variables and Intentions to Attend or Support

In the follow up survey (stage 2), males were more likely to attend a program in the coming year than females (45.5% vs. 40.7%; *X*^2^ = 9.216, *df* = 1, *p* = .027). There was no correlation between intention to attend a program and age. The mean education level was higher for those who intended to participate in a program than for those who did not intend to participate in a program (4.4 vs. 4.1; *t* = 2.101, *df* = 418, *p* = .018, *d* = 0.21). The mean household income level for respondents who said they planned to attend a program during the first summer of the COVID pandemic was higher than for people who said no (3.2 vs. 2.7; *t* = 4.380, *df* = 342, *p* < .001, *d* = 0.48). There were no associations between the level of support for 2020 interpretation programs and gender, age, education, and income.

## Discussion

The purpose of this paper was to investigate how the COVID-19 pandemic affected visitor intentions to participate in personal interpretation programs in Alberta’s provincial parks.

In response to our first research question (RQ1), before the pandemic, only 33.8% of previous non-attendees were likely to attend a personal interpretation program as a result of their visit. In contrast, during the pandemic, only 55.8% of previous attendees and 24.9% of previous non-attendees were planning to attend a program in a park. The most important reasons for not planning to attend a program were avoiding becoming infected by others and infecting others. Lowered intention to attend interpretation programs corresponds with other studies of park visitors during the pandemic. For example, park visitors felt less safe during the pandemic than before, largely due to the lack of adherence to safety measures such as social distancing, wearing masks, and trail etiquette (Budruk et al., 2021).

In response to our second research question (RQ2), respondents who had attended personal interpretation programs in 2019 were more likely to have participated in interpretation programs in the following 12 months than non-attendees. In addition, previous attendees expressed greater intentions to participate in upcoming programs during the pandemic than non-attendees. Other studies have shown that past attendance influences demand for visitor services. On the one hand, participation in interpretive programs was a strong predictor of attendance and interest in future programs in Alberta parks (Cook et al., 2021). Similarly, previous experience at a Korean birdwatching festival was associated with a higher interest in interpretive services (measured by willingness to pay; Lee et al., 2010). On the other hand, in two western Australian parks, repeat visitors reported experiencing a higher level of danger of injury from natural causes than first time visitors (Gstaettner et al., 2022). If reports of danger at these Australian parks apply to visitor perceptions of risk from COVID-19 in Alberta’s parks, we would have found that repeat visitors had lower intentions to participate in interpretation programs than newer visitors.

Also in response to RQ2, recent participation in interpretation programs was associated with greater support for offering personal interpretation programs during the pandemic. While only 41.1% of all respondents said that personal interpretation programs should be offered (indicating yes or most definitely), support was higher among previous attendees (56.4%) than non-attendees (24.4%). Hvenegaard (2017) found strong visitor support for interpretive programs, especially among previous attendees and visitors staying longer in a park. In our study, there were no relationships between support and the demographic variables of gender, age, education, or income.

Regarding specific interpretation programs, respondent support was highest for amphitheater shows, followed by guided hikes, point duties, and family events, likely due to the characteristics of each type. For example, amphitheater shows can maintain spacing if groups spread out in venues that are not filled. Guided hikes could maintain social distancing through group limits and spacing along a trail. Point duties typically involved short interactions between interpreters and family units, while other groups wait their turn at a distance. Support for family events may be due to the ability to keep family units a safe social distance from other groups. Bus tours were not favored, likely due to the requirement to sit close to other attendees in a confined space (Zhang et al., 2021), while the other types of interpretation programs occur outdoors which is preferred by park users during COVID-19 (Bustamante et al., 2022).

In response to our third research question (RQ3), demographic variables partially affected intentions to participate in personal interpretation programs during the pandemic. Even though females had participated more often in interpretive programs before the pandemic than males (consistent with Hvenegaard, 2017; Stern et al., 2011), intentions to participate in programs during the pandemic were higher for males than females. One reason for higher female participation before the pandemic is that females have stronger educational motivations for visiting a park (and therefore, attending an interpretive program) than men (Hvenegaard, 2017). Conversely, during the pandemic, it is possible that risk aversion tendencies among females extends from general activities (Maxfield et al., 2010; Weber et al., 2002) to sporting and outdoor recreation activities (Cary & Stephens, 2023; Randler et al., 2020). Among other demographic variables, intentions to participate were greater for respondents with more education than with less education, and for respondents with larger household income than smaller household income. Taff et al. (2021) also found some inequities in outdoor recreation participation during COVID; participation among ethnically diverse groups declined more than groups identified as being white. However, our respondents’ intentions to participate were not related to age, even though other studies showed that older respondents perceived the pandemic to be more severe and that they were more vulnerable than younger respondents (Pearman et al., 2021; Shen et al., 2021). Support for offering interpretive programs was not associated with any of our measured demographic variables.

## Limitations and Future Research

Regarding limitations, the change in sampling methods (onsite for the first survey and remote sampling for the follow up survey) might have affected respondents’ intentions. This study did not attempt to assess visitor intentions to access non-personal forms of interpretation, and only focused on overnight campers in frontcountry campgrounds (and not day use visitors or backcountry campers). In addition, Alberta Parks now offers new online interpretive programs that provide potential participants with alternative interpretive materials. Finally, at the time of the pandemic, the Alberta Government reduced the budget of Alberta Parks, which affected the level of interpretive programing (Corrigan & Hvenegaard, 2021).

Future research should assess visitor intentions to participate in personal interpretation programs throughout the all stages or waves of the pandemic (D. C. Geng et al., 2021). Use of a common scale (e.g., Zenker et al., 2021) for visitor intentions would allow for comparisons across many jurisdictions and by other user group characteristics, such as nationality or culture (e.g., Shen et al., 2021). Research could evaluate the growth of alternative forms of interpretation (virtual, non-personal), and whether they provide suitable substitutes for visitors (Miller-Rushing et al., 2021). Research should examine other user groups, such as day use visitors and backcountry campers, and also explore which factors affect how visitors develop and act upon their intentions during the pandemic (Jahari et al., 2023; Mateer et al., 2021). Last, researchers should determine which safety measures associated with personal interpretation events visitors prefer, tolerate, and support (Jones et al., 2021; Sánchez-Cañizares et al., 2021).

## Implications for Practice

Parks managers, visitors, and interpreters recognize the value that personal interpretation programs have for visitors and the site (Hvenegaard & Shultis, 2016). While the many benefits provided by non-personal forms of interpretation (Leftridge, 2006) are not addressed in this study, many park visitors expect opportunities for personal interpretation in parks (Schliephack et al., 2013). However, the COVID-19 pandemic reduced park offerings of personal interpretation programs, and possibly the intentions of visitors to participate in these programs.

Results from this study can help interpretive and other park staff design and implement personal interpretation programs that address visitor demand and perceptions of safety during periods of public health concern. Since recent experience (or past familiarity) with personal interpretation programs is correlated with support for and intentions to participate in future programs, promotional efforts will likely be most successful at attracting previous participants. However, there is still support and interest from park visitors who have not recently participated in such programs. Therefore, it is important to communicate to both current and potential visitors about the opportunities available and benefits of interpretive programs, along with pandemic safety measures that have been implemented (Collins et al., 2020). Even though COVID-19 safety protocols, such as mandatory masking and social distancing, are relaxed currently, our study results are relevant because some park visitors still practice these protocols. In addition, both managers and visitors will become concerned, or may anticipate, that future variants of COVID-19 or a different disease may change visitor intentions to participate in park service offerings such as interpretation programs. In conclusion, (1) intent to attend and support for interpretive programs can vary during the pandemic (though many people still support it); (2) clear communication of safety measures is important; and (3) proactive planning for future pandemic events is key to avoid service interruption and to meet visitor demands.
